# Racial and Ethnic Differences in Individuals with Sporadic Creutzfeldt-Jakob Disease in the United States of America

**DOI:** 10.1371/journal.pone.0038884

**Published:** 2012-06-18

**Authors:** Brian S. Appleby, Tonya D. Rincon-Beardsley, Kristin K. Appleby, Mitchell T. Wallin

**Affiliations:** 1 Lou Ruvo Center for Brain Health, Department of Psychiatry and Psychology, Neurological Institute, Cleveland Clinic Foundation, Cleveland, United States of America; 2 Department of Psychiatry and Behavioral Sciences, Division of Geriatric Psychiatry and Neuropsychiatry, Johns Hopkins University School of Medicine, Baltimore, Maryland, United States of America; 3 Biotechnology Studies, School of Arts and Sciences, Advanced Academic Programs, Johns Hopkins University, Baltimore, Maryland, United States of America; 4 Department of Neurology, Veterans Affairs Healthcare System, Washington, District of Columbia, United States of America; 5 Cleveland Clinic Center for Regional Neurology, Cleveland Clinic Neurological Institute, Cleveland, Ohio, United States of America; 6 Department of Neurology, Georgetown University Hospital, Washington, District of Columbia, United States of America; Uppsala University, Sweden

## Abstract

**Background:**

Little is known about racial and ethnic differences in individuals with sporadic Creutzfeldt-Jakob disease (sCJD). The authors sought to examine potential clinical, diagnostic, genetic, and neuropathological differences in sCJD patients of different races/ethnicities.

**Methodology/Principal Findings:**

A retrospective study of 116 definite and probable sCJD cases from Johns Hopkins and the Department of Veterans Affairs Healthcare Systems was conducted that examined differences in demographic, clinical, diagnostic, genetic, and neuropathological characteristics among racial/ethnic groups. Age at disease onset differed among racial/ethnic groups. Non-Hispanic Whites had a significantly older age at disease onset compared to the other groups (65 vs. 60, p = 0.036). Non-Whites were accurately diagnosed more rapidly than Whites (p = 0.008) and non-Hispanic Whites were more likely to have normal appearing basal ganglia on brain magnetic resonance imaging (MRI) compared to minorities (p = 0.02). Whites were also more likely to undergo post-mortem evaluation compared to non-Whites (p = 0.02).

**Conclusions/Significance:**

Racial/ethnic groups affected by sCJD demonstrated differences in age at disease onset, time to correct diagnosis, clinical presentation, and diagnostic test results. Whites were more likely to undergo autopsy compared to non-Whites. These results have implications in regards to case ascertainment, diagnosis, and surveillance of sCJD and possibly other human prion diseases.

## Introduction

Creutzfeldt-Jakob disease (CJD) is a rapidly progressive, fatal, neurodegenerative disease caused by a pathologic and transmissible form of the native prion protein (PrP^Sc^) [1]. Sporadic Creutzfeldt-Jakob disease (sCJD) is the most common form of human prion disease with a worldwide incidence ranging from 0.4 to 2.63 individuals per million people per year [2]–[6]. sCJD often presents with neurological symptoms such as dementia, gait disturbance, myoclonus, visual changes, and motor impairments [7]. Prior epidemiological studies of sCJD in the United States of America (U.S.A) have reported a lower age adjusted incidence rate for Blacks compared to Whites, the reasons for which are unknown [8].

The goal of this study was to further explore demographic and diagnostic differences among race/ethnicity of patients with sCJD. The findings of this study emphasize the importance of including race and ethnicity data in future surveillance and research efforts of human prion diseases.

## Methods

### Objectives

To further investigate demographic and diagnostic differences among races and ethnicities of patients with sCJD.

### Participants

Previously collected data between 1995–2010 from Johns Hopkins (JH) and the Department of Veterans Affairs Health Care System (VHS) were analyzed [9]. Using a standardized collection instrument, clinical data were abstracted from medical records of subjects by two of the authors (BSA and KKA) who have clinical and research experience with human prion diseases (Protocol S1). At least twelve of the subjects had been personally evaluated by one or more of the authors prior to their death. JH is a tertiary medical center that receives many national referrals. The VHS is the largest integrated healthcare system within the U.S.A. and services all of the U.S.A. and occupied territories while providing healthcare to approximately 30% of the veterans’ population [10]. Although the sex ratio is biased towards men (approximately 85% of the population), the racial and ethnic diversity of the VHS reflects that seen in the general population of the U.S.A. [11], [12]. As such, VHS data have been used to assess the incidence and risk factors of other neurological disorders among different races/ethnicities [13]. Hence the sample population is geographically heterogeneous and derived from both general and specialized medical centers.

Only probable and definite cases of sCJD were included in the final analyses. Definite sCJD was determined using previously established criteria [14]. Clinically probable sCJD was determined using recent criteria that include brain magnetic resonance imaging (MRI) data [15]. Only MRI data with diffusion weighted (DWI) and fluid attenuated inversion recovery (FLAIR) sequences were used. When available, MRI images were reviewed by experienced prion disease clinicians and researchers (BSA and KKA). In a minority of cases images were not available and neuroradiology reports were used. Racial and ethnic groups were defined by current U.S.A. Census categories [16] and included non-Hispanic Whites, White Hispanics, and non-Hispanic Blacks or African- Americans. Subjects that were not included in these groups were categorized as “other.”

### Description of Procedures or Investigations Undertaken

Medical records of the aforementioned subjects were reviewed using a standardized abstraction instrument by two investigators (B.S.A. and K.K.A.) (Protocol S1). Information on age and date at disease onset, defined as the time at which the first persistent and progressive symptom occurred during the subject’s course of illness, dates at the time of initial presentation to a healthcare professional, diagnosis of CJD, and date of death were collected. Symptoms and their dates at onset were collected. Cerebrospinal fluid (CSF) 14-3-3 protein, electroencephalogram (EEG), and brain MRI results in addition to neuropathological and genetic data were collected from the medical record or through the assistance of the National Prion Disease Pathology Surveillance Center (NPDPSC, www.cjdsurveillance.com).

### Ethics

This study analyzed previously collected data from JH and the VHS. Data was collected from 1995–2010 and were analyzed following IRB approval from both centers as discussed in a previously published paper [9].

### Statistical Methods

Analyses of variance (ANOVA), t-tests, Mantel-Cox analyses, and Cox proportional hazard models were used for continuous variables and Chi-square analyses were used for categorical variables. Logistic regression analyses were used to control for possible confounding factors. Statistical significance was determined by a p-value ≤0.05. IBM SPSS version 19 statistical software was used for performing analyses.

## Results

116 cases of sCJD were included in the present study. Table 1 lists demographic characteristics of study subjects. Racial and ethnic breakdown within the “other” category included American Indian, mixed race, Black Hispanic, and unspecified. Data did not include Asian subjects as no Asian cases were ascertained in the data collection process.

**Table 1:** Demographic characteristics of study subjects.

Characteristic	Non-Hispanic Whites	Non-Hispanic Blacks	Hispanic Whites	Other^a^	Total
	(n = 100, 86%)	(n = 6, 5%)	(n = 6, 5%)	(n = 4, 4%)	(n = 116, 100%)
Age, mean (SD), y	65 (9.3)^b^	59 (10.9)	64 (9.2)	56 (7.0)	65 (9.4)
Survival time, mean (SD), mo	10.5 (15.4)	10.7 (7.4)	16.2 (22.1)	5 (2.2)	10.6 (15.2)
Center (%)					
*JH*	71 (71)	5 (83)	0 (0)	3 (75)	79 (68)
*VHS*	29 (29)	1 (17)	6 (100)	1 (25)	37 (32)
Male (%)	65 (65)	4 (67)	6 (100)	3 (75)	78 (67)
Definite sCJD (%)	59 (59)	3 (50)	3 (50)	1 (25)	66 (57)

JH = Johns Hopkins, VHS = Veterans Administration Healthcare System, sCJD  =  sporadic Creutzfeldt-Jakob disease.

a Includes American Indian, mixed race, Hispanic Black, and unspecified.

b p = 0.036.

Age at disease onset differed between racial/ethnic groups (Table 1). Although age at disease onset did not differ across all groups in an ANOVA analysis (p = 0.103), non-Hispanic Whites had a significantly older mean age at onset compared to the other groups (65 vs. 60 years) (t-test, t = –2.13, df = 114, p = 0.036). Subjects in the “other” group had the youngest mean age at onset (56 years, SD = 7.0) followed by non-Hispanic Blacks (59 years, SD = 10.9) and Hispanic Whites (64 years, SD = 9.2). Although the “other” group had a much shorter mean survival time (5 months, SD = 2.2), racial/ethnic groups did not differ significantly in disease duration (p = 0.68) (Table 1).

**Figure 1:**
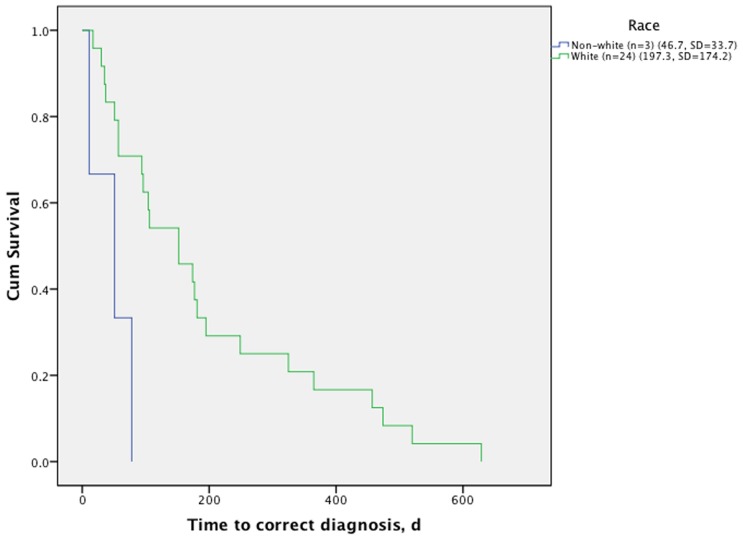
Time to correct diagnosis of sporadic Creutzfeldt-Jakob disease subjects by race/ethnicity. White race includes non-Hispanic and Hispanic Whites (Mantel-Cox, chi-square  = 7.1, p = 0.008).

Time from disease onset to correct diagnosis differed significantly between non-Hispanic and Hispanic Whites (197 days, SD = 174) and non-Whites (46.7 days, SD = 33.7) (Mantel-Cox, chi-square  = 7.1, p = 0.008) (Figure 1). Although there was no statistically significant difference between time from disease onset to initial presentation to a healthcare professional, non-White patients (n = 3) were correctly diagnosed significantly earlier (mean = 46.7 days, SD = 33.7) compared to non-Hispanic and Hispanic Whites (n = 24) (mean = 197.3 days, SD = 174.2) (Mantel-Cox, chi-square  = 7.1, p = 0.008). Differences in mean time to correct diagnosis remained between the two groups (p = 0.03) when age at disease onset was included in a Cox regression proportional hazards model.

The presence of different clinical symptoms did not differ between racial/ethnic groups (Table 2), but time to onset of specific symptoms did vary. Subjects in the “other” category presented with movement disorder symptoms (n = 3, 5 weeks, SD = 2.6) earlier than the other groups (n = 70, 25.6 weeks, SD = 4) in cases when date of symptom onset was known (Mantel-Cox, chi-square  = 4.7, p = 0.03). The non-Hispanic Black (n = 2, 11.5 weeks, SD = 3.5) and “other” racial/ethnic groups (n = 3, 3 weeks, SD = 3) also presented with language impairments earlier in their disease course compared to non-Hispanic Whites (n = 41, 32.7 weeks, SD = 49.3) (Mantel-Cox, chi-square = 7.3, p = 0.03) in cases when date of symptom onset was known.

**Table 2:** Clinical symptoms of subjects throughout the disease course.

Clinical Symptoms (%)	Non-Hispanic Whites	Non-Hispanic Blacks	Hispanic Whites	Other	Total
	(n = 100)	(n = 6)	(n = 6)	(n = 4)	(n = 116)
Cognitive	94 (94)	6 (100)	6 (100)	4 (100)	110 (95)
Cerebellar	82 (82)	4 (67)	5 (83)	3 (75)	94 (81)
Movement disorder	66 (66)	2 (33)	4 (67)	3 (75)	75 (65)
Visual	65 (65)	3 (50)	3 (50)	3 (75)	74 (64)
Myoclonus	61 (61)	5 (83)	5 (83)	2 (50)	73 (63)
Mood	49 (49)	2 (33)	5 (83)	2 (50)	58 (50)
Psychosis	41 (41)	2 (33)	2 (33)	2 (50)	47 (41)
Motor	25 (25)	1 (17)	2 (33)	1 (25)	29 (25)

Cognitive: cognitive decline, executive dysfunction, amnesia, agnosia, apraxia, alexia, language impairment, disorientation, and/or concentration impairment; Cerebellar: ataxia, nystagmus, and/or vertigo; Movement disorder: tremor, chorea, and/or extrapyramidal symptoms (does not include myoclonus); Visual: diplopia, oculomotor palsy, hemianopia, cortical blindness, visuospatial impairment, and/or visual hallucinations; Mood: depression, anxiety, mania, hypomania, emotional lability, and/or apathy; Psychosis: delusions, hallucinations, and/or psychosis not otherwise specified.

Ethnic groups also differed in their time from disease onset to undergoing certain diagnostic tests (Table 3). Members of the “other” group had EEGs performed earlier than the other racial/ethnic groups (p = 0.046, HR = 2.9, 95% CI = 1.0–8.3). An initial EEG was performed at a mean of 7.8 weeks (SD = 5.3) after disease onset in the “other” group compared to 27.1 weeks (SD = 35.7) in the remaining racial/ethnic groups. This finding was no longer statistically significant when age at disease onset was entered into the model (p = 0.051).

**Table 3:** Diagnostic testing data of study sample.

Diagnostic Test Results	Non-Hispanic Whites	Non-Hispanic Blacks	Hispanic Whites	Other	Total
	(n = 100)	(n = 6)	(n = 6)	(n = 4)	(n = 116)
14-3-3 performed (%)^a^	66 (66)	4 (67)	4 (67)	3 (75)	77 (66)
Mean time to 14-3-3 (s.e.), wks	18.6 (2.9)	18 (5.1)	75.5 (54)	6.3 (3.4)	21.4 (4.1)
+14-3-3 (%)^b^	41/60 (68)	2/3 (67)	4/4 (100)	2/2 (100)	49/69 (71)
EEG performed (%)^c^	89/96 (93)	6/6 (100)	4/6 (67)	4/4 (100)	103/112 (92)
Mean # of EEG’s performed	1.6	1.5	1.3	1	1.5
Mean time to EEG (s.e.), wks	28 (4.7)	30 (11)	73 (56)	7.8 (2.7)	30 (5)
PSWC (%)^a^	30/89 (34)	3/6 (50)	1/4 (25)	2/4 (50)	36/103 (35)

EEG = electroencephalogram; PSWC  =  periodic sharp wave complexes.

a Unknown in 9 cases.

b Unknown in 8 cases.

c Unknown in 4 cases.

Brain MRI findings differed between racial/ethnic groups (Table 4). Non-Hispanic Whites (44/95, 46%) were less likely to have hyperintensity of the basal ganglia on.

brain MRI compared to other races/ethnicities (12/15, 80%) (Fisher’s exact test, 2-sided, p = 0.024). This finding remained significant in a binary logistic regression model that included age at disease onset (p = 0.023). There were no statistically significant differences in the prevalence of vascular disease or vascular risk factors (stroke, diabetes mellitus, hypertension, coronary artery disease, or hyperlipidemia) between these two groups.

**Table 4:** Brain magnetic resonance imaging (MRI) characteristics of study subjects.

Area of Hyperintensity	Non-Hispanic Whites	Non-Hispanic Blacks	Hispanic Whites	Other	Total
	(n = 95)	(n = 5)	(n = 6)	(n = 4)	(n = 110)
Mean # of brain MRI’s (SD)	1.8 (0.75)	1.5 (1)	1.5 (0.54)	1.75 (0.5)	1.75 (0.74)
Median time from onset to initial MRI (S.E), wks^a^	10 (1.96)	13 (3.46)	17 (6.12)	5 (4.5)	11 (1.69)
Basal ganglia (%)	44 (46)^b^	4 (80)	5 (83)	3 (75)	56 (51)
Thalamus (%)^c^	15 (16)	1 (20)	1 (17)	2 (50)	19 (17)
Meets cortical criteria (%)^d^	29 (31)	1 (20)	2 (33)	2 (50)	34 (31)
Meets Brain MRI Criteria for sCJD (%)^e^	57 (60)	4 (80)	6 (100)	3 (75)	70 (64)

Brain MRI was not done for 2 subjects and results were unknown in 4 subjects.

a imes could not be calculated for 7 subjects.

b p<0.05.

c 13/15 cases also had basal ganglia hyperintensity.

d Hyperintensity on DWI/FLAIR in 2 or more cortical areas (temporal, parietal, or occipital lobes) [15].

e High signal abnormalities in caudate nucleus and putamen or at least two cortical regions (temporal-parietal-occipital) either in DWI or FLAIR [15].

Although tissue confirmed diagnoses did not differ significantly between groups, non-Hispanic Blacks and “other” racial/ethnic groups were less likely to undergo autopsy (1/10, 10%) compared to non-Hispanic and Hispanic Whites (51/103, 49.5%) (Fisher’s exact test, 2- sided, p = 0.02) (Table 5). JH (16/78, 21%) pursued a more aggressive brain biopsy policy compared to the VHS (2/35, 6%) (Fisher’s exact test, 2-sided, p = 0.054). Full prion protein gene (PRNP) analyses were performed on 45 cases (38.8%) (Table 5). No pathogenic mutations or family history of prion diseases were present in this sample. No statistically significant differences were detected in the codon 129 polymorphism of PRNP or prion protein type (I, II, I and II) between race/ethnic groups (Table 5).

**Table 5:** Neuropathologic and molecular data of study subjects.

Characteristic	Non-Hispanic Whites	Non-Hispanic Blacks	Hispanic Whites	Other	Total
	(n = 100)	(n = 6)	(n = 6)	(n = 4)	(n = 116)
Tissue collected (%)	63 (63)^a^	3 (50)	4 (67)	1 (25)	71 (61)
Autopsy (%)^b^	47 (47)	1 (17)	4 (67)	0	52 (45)
Biopsy (%)	15 (15)	2 (33)	0	1 (25)	18 (16)
*PRNP* analysis performed (%)	39 (39)	2 (33)	3 (50)	1 (25)	45 (39)
*PRNP* codon 129 (%)^c^	MM = 16 (42)	MM = 0	MM = 1 (33)	MM = 1 (100)	MM = 18 (42)
	MV = 15 (40)	MV = 0	MV = 2 (67)	MV = 0	MV = 17 (40)
	VV = 7 (18)	VV = 1 (100)	VV = 0	VV = 0	VV = 19 (18)
Molecular subtype (%)^d^					
MM1	11 (30)		1 (33)	1 (100)	13 (31)
MM2	2 (5)				2 (5)
MM1&2	2 (5)				2 (5)
MV1	6 (16)				6 (14)
MV2	2 (5)		2 (67)		4 (10)
MV1&2	5 (14)				5 (12)
VV1	1 (3)				1 (2)
VV2	4 (11)				4 (10)
VV1&2	2 (5)	1 (100)			3 (7)
MVPSPr	2 (5)				2 (5)

PRNP = prion protein gene, M = methionine, V = valine, PSPr = protease sensitive proteinopathy.

a It was unknown whether tissue was from an autopsy or biopsy in 3 cases.

b Any White race vs. any non-White race (Fisher’s exact test, 2-sided, p = 0.02).

c We could not confirm PRNP codon 129 genotype in 2 cases.

d Defined as PRNP codon 129 genotype and prion protein type(s).

## Discussion

Epidemiological studies are important in the field of human prion diseases given the risk of acquired prion disease and previously recognized genetic factors that affect susceptibility to the disease [17], [18]. Prior epidemiological studies within the U.S. have demonstrated a lower age-adjusted incidence of prion disease in Blacks [8] compared to Whites. Additionally, few cohorts are available to evaluate the morbidity of prion disease in minorities within the U.S.A.

In this study population, minority patients had an earlier age at disease onset compared to non-Hispanic Whites. Non-White patients received an accurate clinical diagnosis more quickly than non-Hispanic and Hispanic Whites. While there was not a statistically significant difference between the race/ethnic groups with regard to disease duration, the “other” group had considerably shorter illness duration (5 months) compared to the other groups. Although this sample’s mean survival time (10.6 months) was longer than what has been described in European studies (7.3 months) [19], it is consistent with prior U.S. studies (8.46–12.62 months) [20]. Moreover, a large European study examining factors that affect survival time noted differences in this statistic between countries [19] that may be influenced by the country’s attitudes towards end of life care [21].

This discrepancy in time from disease onset to diagnosis might be due, in part, to differences in the time of onset of specific symptoms between the race/ethnic groups. Subjects in the “other” category presented with movement disorder symptoms earlier than non-Hispanic White subjects. Perhaps the earlier presence of movement disorder symptoms in these younger subjects aided in making the correct diagnosis more apparent. Similarly, non-Hispanic Black and “other” subjects had an earlier onset of language impairment compared to non-Hispanic Whites. The earlier manifestation of these symptoms may have enabled a more timely diagnosis of sCJD in minorities by clinicians. Additionally, access to medical care within the VHS, a centralized healthcare system with a standardized electronic medical record, in contrast to private healthcare systems or other government programs may have improved detection of symptoms for minorities and/or Hispanic Whites.

Racial/ethnic groups also differed in the time from disease onset to undergoing certain diagnostic tests. Members of the “other” group had EEGs performed earlier than other races/ethnicities, although this was partly influenced by the age of the patient in addition to his or her race/ethnicity.

Initial brain MRI findings also differed between race/ethnic groups. Non-Hispanic White patients were more likely to have normal appearing basal ganglia compared to minority groups. This group had a lower percentage of abnormal brain MRI’s than what would be expected (60% vs. 81%), which may have biased analyses [15]. As brain MRI findings enter into the diagnostic criteria when diagnosing sCJD, a higher rate of abnormal findings in this study’s minority patients may have aided physicians in making a more timely diagnosis in these racial/ethnic groups.

Many prior studies have revealed genetic associations with clinical characteristics of human prion disease. Age at disease onset, survival time, and clinical and neuropathological phenotypes are influenced by PRNP codon 129 polymorphism and prion protein type [22], [23]. The normal distribution of the codon 129 polymorphism varies throughout the world and across ethnic groups [24]. Hence different ethnicities may differ in codon 129 polymorphisms, which could affect age at disease onset, clinical manifestations, and diagnostic test results. Given the small sample size of minorities in the current study, a larger sample of different racial/ethnic groups with genetic and neuropathological data are needed to properly interpret the associations found in this study.

While tissue diagnosis did not differ significantly between race/ethnic groups, non-White subjects were less likely to undergo autopsies. This finding is particularly important when considering reported incidence rates of sCJD that rely heavily on autopsy-derived data. The decreased autopsy rate in minorities could significantly influence the reported incidence of sCJD in this population and result in a falsely lower incidence rate. This is further complicated by the declining autopsy rates in general over the past 5 decades in the U.S.A., which are performed in fewer than 6% of non-forensic deaths [25]. Education provided through clinicians and advocacy groups regarding the importance of and the resources available to perform autopsies on suspected cases of sCJD are crucial to ameliorate the lower autopsy rate in non-Whites. These findings also accentuate the importance of robust clinical sCJD surveillance efforts.

In conclusion, results from this study demonstrate differences in various aspects of sCJD among different race/ethnic groups. Demographic, clinical, and diagnostic differences were ascertained as well as differences in autopsy rates. These findings accentuate the importance of including race and ethnicity data in future studies and surveillance efforts of human prion disease.

### Limitations

Different ethnicities may differ in codon 129 polymorphism which could affect age at disease onset, disease duration, clinical manifestations, and diagnostic test results. Given the small sample size of minorities in the current study, a larger sample of different racial/ethnic groups with genetic and neuropathological data is needed to properly interpret the associations found in this study. Also, because non-White subjects are less likely to undergo autopsies, the decreased autopsy rate in minorities could significantly influence the reported incidence of sCJD in this population and result in a falsely lower incidence rate. All MRI images were not available for standardized analyses and this may have affected these data. No Asians were included in this study and the previously reported low incidence rate of sCJD in Taiwan and Japan (0.63–1.1 individuals per million people) make them an important sample to include in future studies [3], [4]. A prospective study capturing clinically diagnosed and neuropathologically confirmed cases of sCJD would be an ideal study that would maximize reliability.

## Supporting Information

Protocol S1 **Retrospective Creutzfeldt-Jakob disease database clinical entry form.** This form was used by all data collectors (BSA and KKA) to abstract demographic, historical, clinical, diagnostic test results, genetic, and neuropathological data.
(DOC)
