# Supplementary material for: Racial and Ethnic Differences in Individuals with Sporadic Creutzfeldt-Jakob Disease in the United States of America
Source: PLoS One. 2012 Jun 18;7(6):e38884. doi: 10.1371/journal.pone.0038884 (PMC3377728; doi:10.1371/journal.pone.0038884)
Supplement: Protocol S1 — Retrospective Creutzfeldt-Jakob disease database clinical entry form. This form was used by all data collectors (BSA and KKA) to abstract demographic, historical, clinical, diagnostic test results, genetic, and neuropathological data. (DOC) [file pone.0038884.s001.doc]

DEMOGRAPHICS

Sex: O 1 Male O 2 Female O 3 Unknown

Race: O 1 White O 2 Black O 3 Hispanic O 4 Asian O 5 Other:_________

Date of Illness Onset: _ _/_ _/_ _ _ _ Date of Death: _ _/_ _/_ _ _ _

Date of Initial Presentation: _ _/_ _/_ _ _ _

Type of Doctor Initially Presented to:

Initial Dx: □ Dementia, not specified □ Alzheimer’s disease

□ Vascular dementia □ Mixed dementia

□ Lewy body dementia

□ Cognitive disorder, not specified/Mild cognitive impairment □ Frontotemporal dementia □ Prion disease (CJD) □ Stroke

□ Paraneoplastic syndrome □ Depression

□ Other:___________

□ Unknown

Family History:

Hx of dementia? □ Yes □ No □ Unknown

**If yes,** **note # of family members for each dx, age at onset and illness duration**:

______Dementia, unspecified______Parkinson’s ______Alzhemier’s

______Frontotemporal dementia ______Vascular dementia

______Prion disease ______DLB ______Mixed dementia

______Other:_______________________

Psychiatric: ___Depression ___Bipolar ___Anxiety disorder

___Panic disorder ___OCD ___Schizophrenia ___Substance abuse ___Suicides

___Other:_____________

Premorbid Co morbidities:

Hx of Cerebrovascular disease (stroke)? □ Yes □ No □ Unknown

All other Medical/Neurological co-morbidities:

________________________________________________________________________________________________________________________________________________________________________________________________________________________________________________________________________________________________________________________________________________________________________

Hx of Traumatic brain injury? □ Yes □ No □ Unknown

If yes, fill out TBI form

Does the patient have a psychiatric history? □ Yes □ No □ Unknown

Psychosis O 1 Yes O 0 No

Depression O 1 Yes O 0 No

Bipolar disorder O 1 Yes O 0 No

Anxiety disorder O 1 Yes O 0 No

Age of onset of Affective disorder (MDD, BPAD, or anxiety d/o):________

Substance abuse O 1 Yes O 0 No

Specify substance(s):_____________

Other psych:_______________

Possible Modes of Transmission:

History of Tissue Transplantation O 1 Yes O 0 No

If yes, specify: O 1 Dura O 2 Cornea O 3 Other:____________

Date of Transplant: _ _/_ _/_ _ _ _

History of Cadaveric Pituitary Hormones O 1 Yes O 0 No

If yes, date: _ _/_ _/_ _ _ _

History of Any Surgery: □ Yes □ No □ Unknown

1. History of Neurosurgery O 1 Yes O 0 No

If yes, date: _ _/_ _/_ _ _ _ Type:____________________

If yes, date: _ _/_ _/_ _ _ _ Type:____________________

If yes, date: _ _/_ _/_ _ _ _ Type:____________________

2. History of General Surgery □ Yes □ No □ Unknown

If yes, date: _ _/_ _/_ _ _ _ Type:____________________

If yes, date: _ _/_ _/_ _ _ _ Type:____________________

If yes, date: _ _/_ _/_ _ _ _ Type:____________________

If yes, date: _ _/_ _/_ _ _ _ Type:____________________

History of Blood Transfusion O 1 Yes O 0 No O Unknown

If yes, specify dates: date: _ _/_ _/_ _ _ _, _ _/_ _/_ _ _ _, _ _/_ _/_ _ _ _

History of Ingestion of Possible O 1 Yes O 0 No O Unknown

Contaminated Meat Products

If yes, specify type: O 1 Possibly infected BSE beef O 2 Possible Scrapie

O 3 Kuru O 4 Other:_________

Specify first age of consumption:__________

International travel? O 1 Yes O 0 No O Unknown

If yes, specify years: _____ _____ _____

Places: _____ _____ _____

SYMPTOMS (**note approximate date (mm/dd/yyyy) of onset ,** if unknown, note year or “999”)

Neurologic Presented Ever had

Movement disorder ________ _______ (O parkinsonian)

Myoclonus ________ _______

Visual/Oculomotor ________ _______

Cerebellar/ Balance ________ _______

Pyramidal ________ _______

Seizure ________ _______

Akinetic Mutism ________ _______

Speech Disturbance ________ _______

Vertigo ________ _______

Abnormal Sensations ________ _______

Headache ________ _______

Weight Loss(amt lbs) ________ _______

Primitive reflexes: ________ _______ Types:__________________

Hyporeflexic ________ _______ Where:_________________

Hyperreflexic ________ _______ Where:_________________

Babinski ________ _______ Unilateral:___ Bilateral:___

Obtunded ________ _______

Urinary incontinence ________ _______

Dysphagia ________ _______

Other Neuro: ________ _______ Symptom:_______________

Psychiatric Presented Ever had

Depression ________ _______

Apathy ________ _______

Mania/hypomania ________ _______

Mood Lability ________ _______

Dementia/Cog Decl ________ _______

Executive Dysfxn ________ _______

Impaired Memory ________ _______

Impaired Language ________ _______

Delirium/Confusion ________ _______

Poor Concentration ________ _______

Behavior/

Personality change ________ _______

Agitation/Irritability ________ _______

Psychosis NOS ________ _______

Grandiose Delusion ________ _______

Paranoid Delusions ________ _______

Auditory HA ________ _______

Visual HA ________ _______

Tactile HA ________ _______

Olfactory HA ________ _______

Gustatory HA ________ _______

Anxiety ________ _______

Panic ________ _______

Obsessions ________ _______

Compulsions ________ _______

Sleep Disturbance ________ _______

Fatigue ________ _______

Other ________ _______ Symptom:______________

LABORATORY DATA

1. 14-3-3 checked? O 1 Yes O 0 No O 2 Unknown

CSF sample #1:

Result: O 0 Negative O 1 Positive

O 2 Indeterminate

Value (ng/mL):______________(or description if no value)

Lab Facility: O 0 Unknown O 1 Case Western

O 2 Other:______________________

Date: _ _/_ _/_ _ _ _

CSF sample #2:

Result: O 0 Negative O 1 Positive

O 2 Indeterminate

Value (ng/mL):______________(or description if no value)

Lab Facility: O 0 Unknown O 1 Case Western

O 2 Other:______________________

Date: _ _/_ _/_ _ _ _

CSF sample #3:

Result: O 0 Negative O 1 Positive

O 2 Indeterminate

Value (ng/mL):______________(or description if no value)

Lab Facility: O 0 Unknown O 1 Case Western

O 2 Other:______________________

Date: _ _/_ _/_ _ _ _

2. CSF Enolase Checked? O 1 Yes O 0 No

Result: O 0 Negative O 1 Positive

O 2 Indeterminate

Value (ng/mL):______________

Date: _ _/_ _/_ _ _ _

(If further, enolase levels or other CSF levels were checked (i.e. t-tau), pls record info as listed above in following space:_____________________________________)

3. EEG checked? O 1 Yes O 0 No O 2 Unknown

**(provide copies on CD if possible)**

EEG #1:

Result: O 1 Positive O 0 Negative If positive:

Slowing O 1 Yes O 0 No

Symmetry O 1 Right O 2 Left O 3 Bilateral

FIRDA O 1 Yes O 0 No

PLEDS O 1 Yes O 0 No

Triphasics O 1 Yes O 0 No

Symmetry O 1 Right O 2 Left O 3 Bilateral

Date: _ _/_ _/_ _ _ _

EEG #2:

Result: O 1 Positive O 0 Negative If positive:

Slowing O 1 Yes O 0 No

Symmetry O 1 Right O 2 Left O 3 Bilateral

FIRDA O 1 Yes O 0 No

PLEDS O 1 Yes O 0 No

Triphasics O 1 Yes O 0 No

Symmetry O 1 Right O 2 Left O 3 Bilateral

Date: _ _/_ _/_ _ _ _

EEG #3:

Result: O 1 Positive O 0 Negative If positive:

Slowing O 1 Yes O 0 No

Symmetry O 1 Right O 2 Left O 3 Bilateral

FIRDA O 1 Yes O 0 No

PLEDS O 1 Yes O 0 No

Triphasics O 1 Yes O 0 No

Symmetry O 1 Right O 2 Left O 3 Bilateral

Date: _ _/_ _/_ _ _ _

4. MRI checked? O 1 Yes O 0 No O 2 Unknown

**(provide copies on CD if possible)**

MRI #1:

Result: O 1 Positive O 0 Negative

If positive, areas of hyperintensity:

Caudate/Putamen O 1 Yes O 0 No

Laterality: O 1 Right O 2 Left O 3 Bilateral

GPi O 1 Yes O 0 No

Laterality: O 1 Right O 2 Left O 3 Bilateral

Thalamus O 1 Yes O 0 No

Laterality: O 1 Right O 2 Left O 3 Bilateral

Frontal Cortex O 1 Yes O 0 No

Hemisphere: O 1 Right O 2 Left O 3 Bilateral Temporal Cortex O 1 Yes O 0 No

Hemisphere: O 1 Right O 2 Left O 3 Bilateral

Parietal Cortex O 1 Yes O 0 No

Hemisphere: O 1 Right O 2 Left O 3 Bilateral Occipital Cortex O 1 Yes O 0 No

Hemisphere: O 1 Right O 2 Left O 3 Both

Other:____________ O 1 Yes O 0 No

Hemisphere: O 1 Right O 2 Left O 3 Both

Presence of Atrophy: O 1 Yes O 0 No

Symmetry O 1 Right O 2 Left O 3 Bilateral

Imaging Modality Revealing Intensities:

T1 O 1 Yes O 0 No

T2 O 1 Yes O 0 No

DWI O 1 Yes O 0 No

FLAIR O 1 Yes O 0 No

Date: _ _/_ _/_ _ _ _

MRI #2:

Result: O 1 Positive O 0 Negative

If positive, areas of hyperintensity:

Caudate/Putamen O 1 Yes O 0 No

Laterality: O 1 Right O 2 Left O 3 Both

GPi O 1 Yes O 0 No

Laterality: O 1 Right O 2 Left O 3 Bilateral

Thalamus O 1 Yes O 0 No

Laterality: O 1 Right O 2 Left O 3 Bilateral

Frontal Cortex O 1 Yes O 0 No

Hemisphere: O 1 Right O 2 Left O 3 Both

Temporal Cortex O 1 Yes O 0 No

Hemisphere: O 1 Right O 2 Left O 3 Both

Parietal Cortex O 1 Yes O 0 No

Hemisphere: O 1 Right O 2 Left O 3 Both

Occipital Cortex O 1 Yes O 0 No

Hemisphere: O 1 Right O 2 Left O 3 Both

Other:____________ O 1 Yes O 0 No

Hemisphere: O 1 Right O 2 Left O 3 Both

Presence of Atrophy: O 1 Yes O 0 No

Symmetry O 1 Right O 2 Left O 3 Bilateral

Imaging Modality Revealing Intensities:

T1 O 1 Yes O 0 No

T2 O 1 Yes O 0 No

DWI O 1 Yes O 0 No

FLAIR O 1 Yes O 0 No

Date: _ _/_ _/_ _ _ _

MRI #3:

Result: O 1 Positive O 0 Negative

If positive, areas of hyperintensity:

Caudate/Putamen O 1 Yes O 0 No

Laterality: O 1 Right O 2 Left O 3 Both

GPi O 1 Yes O 0 No

Laterality: O 1 Right O 2 Left O 3 Bilateral

Thalamus O 1 Yes O 0 No

Laterality: O 1 Right O 2 Left O 3 Bilateral

Frontal Cortex O 1 Yes O 0 No

Hemisphere: O 1 Right O 2 Left O 3 Both

Temporal Cortex O 1 Yes O 0 No

Hemisphere: O 1 Right O 2 Left O 3 Both

Parietal Cortex O 1 Yes O 0 No

Hemisphere: O 1 Right O 2 Left O 3 Both

Occipital Cortex O 1 Yes O 0 No

Hemisphere: O 1 Right O 2 Left O 3 Both

Other:____________ O 1 Yes O 0 No

Hemisphere: O 1 Right O 2 Left O 3 Both

Presence of Atrophy: O 1 Yes O 0 No

Symmetry O 1 Right O 2 Left O 3 Bilateral

Imaging Modality Revealing Intensities:

T1 O 1 Yes O 0 No

T2 O 1 Yes O 0 No

DWI O 1 Yes O 0 No

FLAIR O 1 Yes O 0 No

Date: _ _/_ _/_ _ _ _

5. Biopsy O 1 Yes O 0 No

If yes:

Spongiform changes on autopsy: O 1 Yes O 0 No

Immunostaining performed? O 1 Yes O 0 No

Results: O 1 Positive O 0 Negative WB performed? O 1 Yes O 0 No

___PrPsc 27-30 ___PrPsc 7-8

___Other:____________________

Molecular subtype:

codon 129 polymorphism: ___MM ___MV ___VV

other polymorphism: ___________________________

Prion protein type ___type 1 ___type 2 ___type 1 and 2

6. Autopsy O 1 Yes O 0 No

If yes:

Spongiform changes on autopsy: O 1 Yes O 0 No

Immunostaining performed? O 1 Yes O 0 No

Results: O 1 Positive O 0 Negative WB performed? O 1 Yes O 0 No

___PrPsc 27-30 ___PrPsc 7-8

___Other:____________________

7. Molecular subtype:

codon 129 polymorphism: ___MM ___MV ___VV

other polymorphism: ___________________________

Prion protein type ___type 1 ___type 2 ___type 1 and 2

___PSPr

PRNP mutation : O 1 Yes O 0 No

If yes, type (include mutation, PrPsc type, and codon 129 polymorphism

on the mutant **and** non-mutant alleles):____________________________

If neuropath done at the NPDPSC, case #:________________

Please provide the following lab work results:

1. ABG or HCO3 level done on the day of the CSF 14-3-3

2. All cholesterol tests (before and after illness)

3. All thyroid function tests (before and after illness)

4. All serum iron studies (before and after illness)

5. Medication forms

6. TBI form if pertinent

7. Copies of any Neuropsychological testing with dates (including bedside MMSE)

8. Copies of studies on CD if possible

Notes:__________________________________________________________________________________________________________________________________________________________________________________________________________________
